# Intra- and inter-task reliability of spatial attention measures in healthy older adults

**DOI:** 10.1371/journal.pone.0226424

**Published:** 2019-12-23

**Authors:** Gesine Märker, Gemma Learmonth, Gregor Thut, Monika Harvey

**Affiliations:** 1 Centre for Cognitive Neuroimaging, Institute of Neuroscience and Psychology, University of Glasgow, Glasgow, United Kingdom; 2 School of Psychology, University of Glasgow, Glasgow, United Kingdom; University of Exeter, UNITED KINGDOM

## Abstract

At present, there is a lack of systematic investigation into intra- and inter-task consistency effects in older adults, when investigating lateralised spatial attention. In young adults, spatial attention typically manifests itself in a processing advantage for the left side of space (“pseudoneglect”), whereas older adults have been reported to display no strongly lateralised bias, or a preference towards the right side. Building on our earlier study in young adults, we investigated older adults, aged between 60 to 86 years, on five commonly used spatial attention tasks (line bisection, landmark, grey and grating scales and lateralised visual detection). Results confirmed a stable test-retest reliability for each of the five spatial tasks across two testing days. However, contrary to our expectations of a consistent lack in bias or a rightward bias, two tasks elicited significant *left* spatial biases in our sample of older participants, in accordance with pseudoneglect (namely the line bisection and greyscales tasks), while the other three tasks (landmark, grating scales, and lateralised visual detection tasks) showed no significant biases to either side of space. This lack of inter-task correlations replicates recent findings in young adults. Comparing the two age groups revealed that only the landmark task was age sensitive, with a leftward bias in young adults and an eliminated bias in older adults. In view of these findings of no significant inter-task correlations, as well as the inconsistent directions of the observed spatial biases for the older adults across the five tested tasks, we argue that pseudoneglect is a multi-component phenomenon and highly task sensitive. Each task may engage slightly distinct neural mechanisms, likely to be impacted differently by age. This complicates generalisation and comparability of pseudoneglect effects across different tasks, age-groups and hence studies.

## Introduction

With a growing senior population, understanding healthy cognitive ageing is imperative for identifying possible markers of cognitive decline. Yet, to date, little is known about the impact of ageing processes on spatial attention, and its progression over the lifespan. Moreover, current research has offered little insight into the neural correlates underpinning spatial attention in the healthy ageing brain. Thus far, research investigating visuospatial attention in healthy, predominantly young to middle aged participants, describes an attention asymmetry towards the left visual space, termed “pseudoneglect” [1]. In pseudoneglect, young adults show an attention asymmetry, typically displaying a leftward spatial bias, when asked to estimate the veridical centre of a centrally presented line in the line bisection task [2]. This spatial bias towards the left side of space is generally interpreted as resulting from an asymmetrical distribution of spatial attention resources, with a right hemisphere dominance over the left hemisphere that favours the left visual field of space when allocating spatial attention [1–3]. In contrast, patients with neglect (typically resulting from right hemisphere stroke) show the opposite pattern: a large rightward spatial attention bias due to perception deficits in the contralesional side of visual space [4]. There has thus been an implicit assumption that the presence of pseudoneglect equates to healthy cognitive performance in the spatial attention domain. In addition, across the lifespan, older adults have been thought to show a reduction of this lateralised spatial attention, resulting in either a reduced or even reversed spatial bias to the right side of space, comparable to patients suffering from hemispatial neglect [5,6]. However, the evidence paints a mixed picture, with some recent studies reporting maintained *leftward* biases into older age e.g. [7,8]. As such, the premise of a rightward shift occurring in all older adults, and across all spatial attention measures, is likely to be too simplistic.

### Five commonly used spatial tasks

Recently Learmonth et al. [9,10] investigated the inter- and intra- task reliability of five commonly used measures of spatial attention in young adults: the Landmark task (LM), a computerised Manual Line Bisection task (MLB), the Greyscale task (GREY), the Gratingscale tasks (GRA), and the Lateralised Visual Detection task (LVD). Although all 5 tasks are commonly used to quantify the direction and magnitude of lateralised spatial attention biases, the tasks all require different types of perceptual judgements to be performed. The LM and MLB tasks require a judgement of whether the left relative to the right segment of a horizontal line is longer. The GREY task requires an indication of whether the left or the right side of space is darker. For the GRA task participants judge whether the left or right side of space contains more sinusoidal gratings. Finally, the LVD task assesses whether small targets are perceived more accurately in the left or the right side of space. We reported a lack of correlation between these five spatial tasks in young adults [9,10], a finding described previously in studies probing spatial biases using line bisection, chimeric faces, Muller-Lyer illusion lines and variations of the greyscales task [11–13] and we suggested that this strongly implicates spatial attention as a multi component phenomenon. Importantly though, we found moderate-to-strong intra-task correlations of the 5 tasks across different testing days, suggesting each of them to be a reliable measure of spatial bias on their own. The landmark and line bisection task displayed leftward biases (pseudoneglect) across both days, but no consistent significant directional effects emerged for the three other tasks.

#### Spatial attention in older adults

It recently become apparent that the direction of the spatial biases in cognitively healthy older participants are even less consistent across the wider literature [14] and below we briefly present findings for the 5 most commonly used spatial tasks described above.

#### Manual line bisection task

This task is generally assessed by asking participants to mark the centre of a line presented in front of them. Line bisection tasks have yielded very varied results with a shift of bias towards the right side of space in older adults compared to young adults [15–19], as well as a lack of age related differences in spatial bias between young and older adults, with older adults showing no directional bias at all [20–23]. Moreover, a more recent study by Brooks et al.[8] identified a maintained leftward spatial bias, and hence reporting pseudoneglect in both young and older groups.

#### Landmark task

Similarly, for the landmark task, participants typically have to indicate which side of a pre-transected centrally presented line appears to be shorter (or longer). Results have ranged from reduced to reversed spatial biases (i.e. rightward shifts) in older adults. For instance, while young adults have shown a leftward bias, some studies have reported an attenuation of this left bias with age [24–26], while others found no age differences for group level spatial biases despite a trend towards the right side of space in older adults compared to young adults [23,27].

#### Greyscale task

Other tasks probing spatial lateralisation involving target features have been the Greyscale [13] and the procedurally similar Gratingscale [28] task. For the Greyscale task, participants typically indicate which one of two horizontally parallel rectangular bars is *darker* overall, while for the Gratingscale task, participants judge which of two horizontally parallel presented horizontal rectangular bars contains more *thin stripes* overall.

For both tasks, pseudoneglect has been reported in young adults [13,28]. With regard to spatial lateralisation in older adults, results are sparse and tend to focus on patient groups with hemispatial neglect. So far, only two studies have reported on the performance of older adults on the greyscale task, both suggesting a reliable leftward bias in older adults (pseudoneglect) [7,29]. Interestingly, investigating seven age groups, Friedrich et al. [7] highlighted that all age groups showed a leftward bias, with the strongest leftward bias in the oldest age group (80–89 years), as compared to the youngest age group (18–29 years). This shows further evidence for leftward biases with increased age rather than a reduced pseudoneglect pattern.

#### Gratingscale task

The Gratingscale task has not yet been investigated in cognitively healthy older adults. Niemeier et al.[28] showed that young participants were more likely to judge the left visual side of space as higher-frequency patterned if the side included a portion of “thin stripes”. However, Learmonth et al. [9,10] reported no spatial bias in young adults when judging high spatial frequencies over multiple days.

#### Lateralised visual detection task

In the lateralised visual detection task, participants have to respond when they see a small target appearing on their left or right side. Varying spatial asymmetry has also been reported for this task, with a leftward bias in young adults [30,31], or an absence of this bias in young adults when targets were not titrated [9,10]. Older adults have shown no spatial bias to either side of space, even with titrated targets [31].

### Aims and hypotheses

Given this lack of consensus of biases shown in older adults in particular, it is important to investigate spatial attention in an older adult population more systematically. To allow for an informed assessment of possible underlying neural plasticity in older participants, as well as to potentially identify diagnostic markers of pathological visuospatial biases, we wanted to establish spatial biases across the 5 tasks described above in a single older adult sample in the first instance. As the majority of spatial attention research so far has focused on young adults (reviewed in [9,10]), the aims we addressed in this paper are threefold: we wanted to 1) understand the directionality of spatial biases in healthy older adults, 2) investigate the stability of these biases over time, and 3) assess their replicability across different tasks. Taking previous findings into consideration [9,10], we tested the intra- and inter-task reliability of five commonly used measures of spatial attention in an ageing population, namely: 1) Manual Line Bisection 2) Landmark, 3) Greyscales, 4) Gratingscales, and 5) the Lateralised Visual Detection task. It was predicted that older adults would show weaker correlations in terms of their spatial attention bias across different testing sessions, similar to our previous results using the lateralised visual detection task [9,10]. However, a correlation between testing sessions of the spatial tasks in older adults would provide evidence that each employed task is a reliable and sensitive measure, even in an older population. As previous results have been mixed, this study is expected to give valuable insights into the accuracy and sensitivity (within and across) of each of these measurements, and ultimately guide researchers and clinicians into choosing the most sensitive task to assess spatial bias across the life span.

## Methods

### Participants

Thirty-seven cognitively healthy older adults aged between 60 to 86 years were tested (19 Females, M = 71 years; S.D. = 6.05). The study received ethical approval from the University of Glasgow, College of Science and Engineering Ethics Committee, and participants gave written informed consent before participation.

#### Pre-screening measurements

All participants were right handed and had normal or corrected-to-normal vision, as per Snellen chart presented at a viewing distance of 280 cm. Participants were pre-screened for possible visual field changes and detection accuracy of small stimuli with a short computerised assessment [27]: Over 36 different positions, a small black dot (10 x10 pixel) appeared for 150ms. Participants were instructed to press the spacebar if they perceived the dot anywhere on the screen, while fixating on the cross in the middle of the screen. The 36 locations extended to 12.0° visual angle (VA) from fixation along the vertical axes and 16.06 ° VA along the horizontal axis. A total of 72 trials were presented (36 locations x2) including 24 ‘catch’ trials where responses were withheld. No participants were excluded based on this visual acuity screening. Furthermore, participants were screened for mild cognitive decline with the Montreal Cognitive Assessment test (MOCA) [32] and all showed normal performance (*M* = 28.61; S.D. = 1.06).

### Procedure

The procedure was identical to Learmonth et al. [10]. However, a pilot phase in 3 older adults highlighted that the LVD task used in Learmonth et al. [9,10] was too perceptually challenging for older adults, and it was thus adapted for this study (see LVD task below). The study was conducted over two separate sessions (a minimum of 24 hours apart) lasting around 1.5 hours (short breaks included). Participants were asked to rate their subjective alertness (from 0 = almost asleep to 100 = fully alert) on a linear scale before and after each session. They were seated in a dimly lit room in front of a computer screen. The viewing distance was kept constant at 60cm from the screen using a chin rest. Each participant completed five spatial tasks: 1) Manual Line Bisection (MLB), 2) Landmark task (LM), 3) Greyscale task (GREY) 4) Gratingscale task (GRA), 5) Lateralised Visual Detection Task (LVD). Test order was counterbalanced across the participants to control for task-order effects. On both testing days, the task order was kept identical for each participant. Each task was introduced with written and verbal instructions and a practice trial of around 20 trials. The blocks lasted roughly 5 minutes each and allowed for a break afterwards. (MBL and LM data of 40 participants have been described in Learmonth, Märker, et al. [23].

### Stimuli

E-Prime 2.0 (Psychology Software Tools Inc., Pittsburgh, PA) was used to present the Stimuli and obtain measures of reaction time and accuracy. The study was executed on a Dell Precision 380 PC and a 19” Dell 1908FP Ultra Sharp LCD flat screen monitor, with a 1280x1024 pixel resolution. One pixel measured approximately 0.29mm^2^. The five spatial tasks described in Learmonth et al. [10](identical in method and measures) were used, with only the viewing distance shortened from 70 to 60cm to accommodate the older sample. The LVD task was also slightly modified to meet the requirements of this older adult sample (see task description below).

#### Manual line bisection task (MLB)

**Fig 1:**
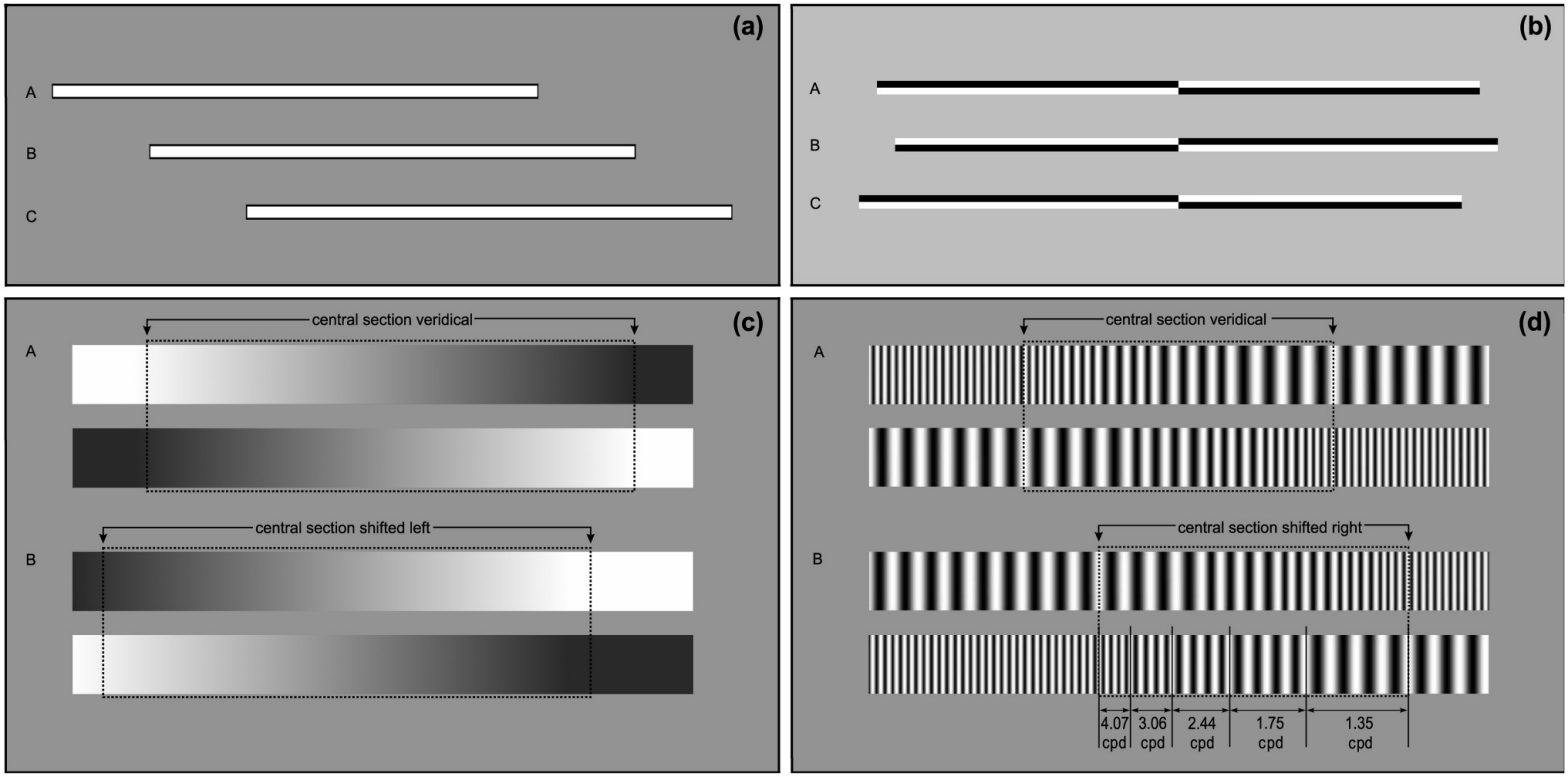
Schematic representation of the stimuli. Examples of the (a) manual line bisection (MLB), (b) landmark (LM), (c) greyscales (GREY) and (d) gratingscales (GRA) stimuli. See [10] PLOS ONE https://doi.org/10.1371/journal.pone.0138379.g001.

Participants were asked to indicate the horizontal centre of a line with the mouse cursor. They completed (108 trials) in which a horizontal white line (805x 15 pixels / 23. 5cm x 0.4cm with a 22.16 x 3.81° visual angle, (VA)) was presented for a maximum of 6 seconds on a grey background (Fig 1a). The position of the line varied laterally on a trial by trial basis, presented at 9 different positions repeated for 12 times along the horizontal axis to the left and to the right of veridical centre (0 = centred, and 40, 80, 120 and 160 pixels, 1.01, 2.02, 3.03, 4.04 ° VA). The mouse pointer appeared at the top centre of the screen (screen co-ordinates: X = 640, Y = 40 pixels; 16.06° above fixation) at the start of each trial and was dragged down by the participant to bisect the line using the left-click of the mouse as accurately as possible. A response triggered the onset of a new trial, with the stimulus appearing after 1000ms. If no response was given, the next trial started after 6 seconds had elapsed.

#### Landmark task (LM)

In each trial, participants were instructed to indicate which side of a pre-transected centrally presented line was shorter using the ‘v’ (left) or ‘b’ (right) keys [2,10,33]. The trial started with a central fixation cross (15x15 pixels; 0.38° VA) for 1000ms and the stimulus appeared for 150ms. A forced-choice response was required in order to move to the next trial. The stimuli consisted of horizontal 100% black and white Michelson contrast lines (800 x14 pixels; 23.5cm x 0.4cm; 20.01 x 0.35° VA). The shading of the upper left and lower right was randomised, with half of the trials involving a dark upper left/lower right, and half lower left/upper right (see Fig 1b). The line was vertically transected at the veridical centre of the screen. The asymmetry of the line varied across trials, resulting in 17 different stimuli with 8 repetitions. The left and right sides of the most asymmetric lines differed by 24 pixels (0.6° VA). Stimuli varied in size, with a decrease of 3-pixels (0.08° VA) per stimulus. One final stimulus involved lines where the left and right sides were of equal length.

#### Greyscale task (GREY)

Participants were instructed to indicate which of two horizontally parallel rectangular bars were darker overall [10,29]. The bars were gradually shaded from black to white until 100% luminance in black on one side and white on the other side was reached, resulting in a smooth luminance gradient. One of the bars was then flipped along the vertical axis so that the lower bar was shaded from black to white in the inverse direction relative to the upper bar (Fig 1c) To allow for an analysis method of psychometric curve widths, a “window of interest” which spanned 640 pixels (80% if the line length, 16.06° VA) was shifted along the horizontal axis to create the stimuli. A total of 17 different stimuli were used, were each window was shifted in 10- pixel increments (0.25 VA) to either side of space (800 x 100 pixels (approximately 23.5cm x 2.9cm; 20.01 x 2.53° VA) with a distance of 41 pixels (1.04°) between the bars. Prior to each trial, a fixation cross was presented for 1000ms, followed by the stimulus for 150ms, and a key press choice indicating if the upper or lower bar was darker overall, with participants using the “upwards arrow” or the “downwards arrow” with their right hand. The maximum deviation from symmetry differed by 80 pixels (2.02°; -10% or +10% of total length) and the remainder of the bar was filled in with black or white. The block consisted of 136 trials with 17 Stimuli repeated 8 times, half of them with the upper left and lower right black and vice versa.

#### Gratingscale task (GRA)

Participants had to indicate via key press (up or down arrow) which horizontal rectangular had more thin stripes over all: the top bar or the parallel presented bottom bar [10,28]. One block consisted of 136 trials with 17 Stimuli repeated 8 times. A fixation cross appeared for 1000ms followed by the stimulus for 150ms, and a response which triggered the next trial. The stimuli were identical to Learmonth et al. [10] (see for stimuli details). The rectangular bar was sine-wave grated with high-frequency grating (35 pixels per cycle; 1.15 cycles per degree of visual angle (cpd)) at one end and low-frequency (11 pixels per cycle; 3.49 cpd) at the other end (Fig 1d). Half of the stimuli had high frequency grating on the upper left and lower right side, and the other half had higher grating on the upper right and lower left side. A central segment of 400 pixels (50% of the total length, 10.08°) was shifted in 12-pixel (0.30°) instalments to either the left or right side to compile 17 different stimuli with the maximum deviation from symmetry at 96 pixels (2.43°; -12% or +12% of total length), and the rest filled with high or low frequency pattern to continue the gradient pattern. The central segment consisted of 5 different spatial frequencies, with 4 sine wave cycles per frequency. They ranged from low spatial frequency = 35 pixels per cycle, through 26, 19, 14 pixels per cycle. The highest frequency consisted of 10 pixels per cycle (i.e. the number of pixels per cycle reduced by a factor of approximately x 0.74) (identical to Learmonth et al. [9,10].

#### Lateralised visual detection task (LVD)

Participants were instructed to indicate whether they saw a small target appearing on their left or right side, with a key press (“v” for left, “b” for right) or no response when no dot appeared (catch trial) [10,30]. A fixation cross appeared for 1000ms followed by the stimulus for 40ms. Participants had to make a response within 1750ms before the next trial started, in order to allow “catch trials” and false negatives. The stimuli consisted of a small target presented either to the left (-145mm; -13.78° VA) or right visual field (+145mm; +13.78° VA)., In contrast to the young sample, the current stimuli consisted of 10 different sizes (1x2, 2x2, 2x3, 3x3, 3x4, 4x4, 4x5, 5x5, 5x6, 6x6 pixels; ranging from 0.03 x 0.05° VA to 0.13 x 0.15° VA), to accommodate the greater variability in detection sensitivity in the older adults [31]. The block consisted of 126 trials for the ten different dot sizes (6 left and 6 right for each of the 10 stimulus sizes, i.e. 60 total targets), plus 6 catch trials. In Learmonth et al. [9,10], young adults were presented with stimuli of 5 different sizes only (1x2, 2x2, 2x3, 3x3, 3x4 pixels resulting in 132 targets).

### Analyses

#### Analysis of PSE and curve widths

The point of subjective equality (PSE) was analysed for the five tasks in order to estimate the magnitude of a spatial bias. Results were then transformed into % of total line length, relevant to the stimuli. The Landmark (LM), Greyscales task (GREY) and Gratingscale (GRA) were analysed in a similar manner. A percentage score was calculated separately for each of the 17 stimuli, where the subject perceived the stimulus to be either longer (LM) / darker (GREY) / have more “thin stripes” (GRA) on the right side of space.

Following this, the data were plotted as psychometric curves (17 stimuli vs percentage of trials where target was judged to be on the right) per individual and per task, and psychometric functions were fitted to this data using the curve fitting toolbox for Matlab [34] to calculate the point of subjective equality (PSE) and curve widths. The psychometric function used the cumulative logistic function described by the equation:

$$f(\mu ,x,s)=1/(1+\text{exp}\left(\frac{x-\mu }{s}\right))$$

In this function, *μ* is the point on the x-axis that equates to 50% left and 50% right-response rate, *x* represents the transector locations and *s* describes the psychometric curve width. The PSE and curve widths were then converted to a percentage of the total line length. Specifically, curve widths indicate an individual’s precision on the task. A narrow curve width can be interpreted as a good performance on the task.

For the MLB task, the subjective midpoint was calculated by subtracting the x- co-ordinate, obtained through the mouse click, from the co-ordinate from the veridical centre of the line. The mean bias and standard deviation for each individual indicated the overall spatial bias for this task.

#### Analysis of D’Prime (d’) and PSE in the LVD task

As per Learmonth et al. [9,10] the LVD task was analysed in two ways: a) D-prime (d’) to determine the visual detection sensitivity, b) fitting a psychometric function to the data (PF 50%).

For the D-prime (d’) calculation we used visual detection sensitivity. *D’* was calculated using the function:

$$d^{\prime }=z(Hits)-z(False\;Alarms)$$

where *z* represents the z-score for each side of space. “Hits” represent the percentage accuracy for each target side (i.e. targets perceived correctly on the left or right side of space), subtracted by the number of “false alarms” (in response to catch trials). Larger *d*’ scores represent a greater sensitivity for detecting stimuli relative to false positives. A *d’* lateralisation index was then calculated by subtracting Left visual field (VF) *d’* from Right VF *d’*.[10]

Similar to the analysis of the LM, GRA–and GREY tasks, a psychometric function was fitted to the 10 stimulus sizes of the LVD task separately for each visual field, and PSE and curve widths were calculated. The stimulus sizes were labelled 1–10, with 1 = the smallest (1x2 pixel) target up to 10 = the largest (6x6 pixel) target. Thus, a small PSE of 1.5 indicates a relatively good visual detection accuracy, placing the 50% accuracy (PF 50%) between the 1x2 and 2x2 target size. A measure of lateralised spatial bias was then calculated by subtracting the Right VF PSE from the Left VF PSE. The PF 50% and *d’* methods were found to correlate on both testing days (Day 1: r = 0.884, p<0.001; Day 2: r = 0.965, p<0.001; Mean Days 1+2: r = 0.937, p<0.001).

#### Outlier detection and winsorized means

Rather than excluding participants due to individual outliers, the group-level spatial biases of each testing day were winsorized. Firstly, the individual spatial biases per day and spatial tasks where screened for outliers that exceeded a spatial bias above 3 x the standard deviation of the group level mean. Once a spatial task was identified to have such outliers, the whole sample of that testing day (37 individual biases) was winsorized to adjust the spatial biases. The winsorized mean was calculated by replacing 20% of the 37 individual biases. The smallest and largest spatial biases were replaced with the values closest to them [35]. There were no outliers identified for the LM and MLB tasks so these remained untrimmed. For the GREY, GRA and LVD tasks 20% winsorized (modified) means were used. For the average bias across both testing days (for each of these 3 spatial tasks) the original (unmodified) means was calculated and then winsorized by 20%. In addition, one participant was excluded from the LVD task due to difficulty in detecting even the largest targets, despite passing the initial visual screening tests and the task modifications that were made (PSE = -22). Nonetheless this participant was not excluded from the other 4 tasks. This resulted in 37 participants for the LM, MLB, GREY and GRA tasks, and 36 participants for the LVD task, maintaining cross-task comparability as much as possible.

## Results

### Subjective alertness

Performance of a 2x2 analysis of variance (ANOVA) (TIME: pre- vs post- experiment x Day: Day 1 and Day 2) revealed that participants showed reduced subjective alertness after the experiment (pre- test Day 1: M = 84.7%; S.D. = 11.17 vs. Post-test Day 1: M = 77.97%; S.D. = 13.09 and pre–test Day 2:M = 87.32%; S.D. = 10.13 vs. Post-test Day 2: M = 81.05%; S.D. = 10.31), Main effect of Time: F(1, 36) = 44.11, p = .001. There was also a main effect of Day: F(1,36) = 4.61, p = .04, with participants more alert overall in the second testing session (Day 1: M = 81.38%; S.D. = 11.52 vs. Day 2: M = 84.18%; S.D. = 9.37), Post Day1 vs Day 2 (t(36) = -2.15, p = .04). Importantly, there was no interaction between Time and Day of testing, with no indication of a larger deterioration of alertness on one day over the other.

### Visual acuity screening

**Fig 2:**
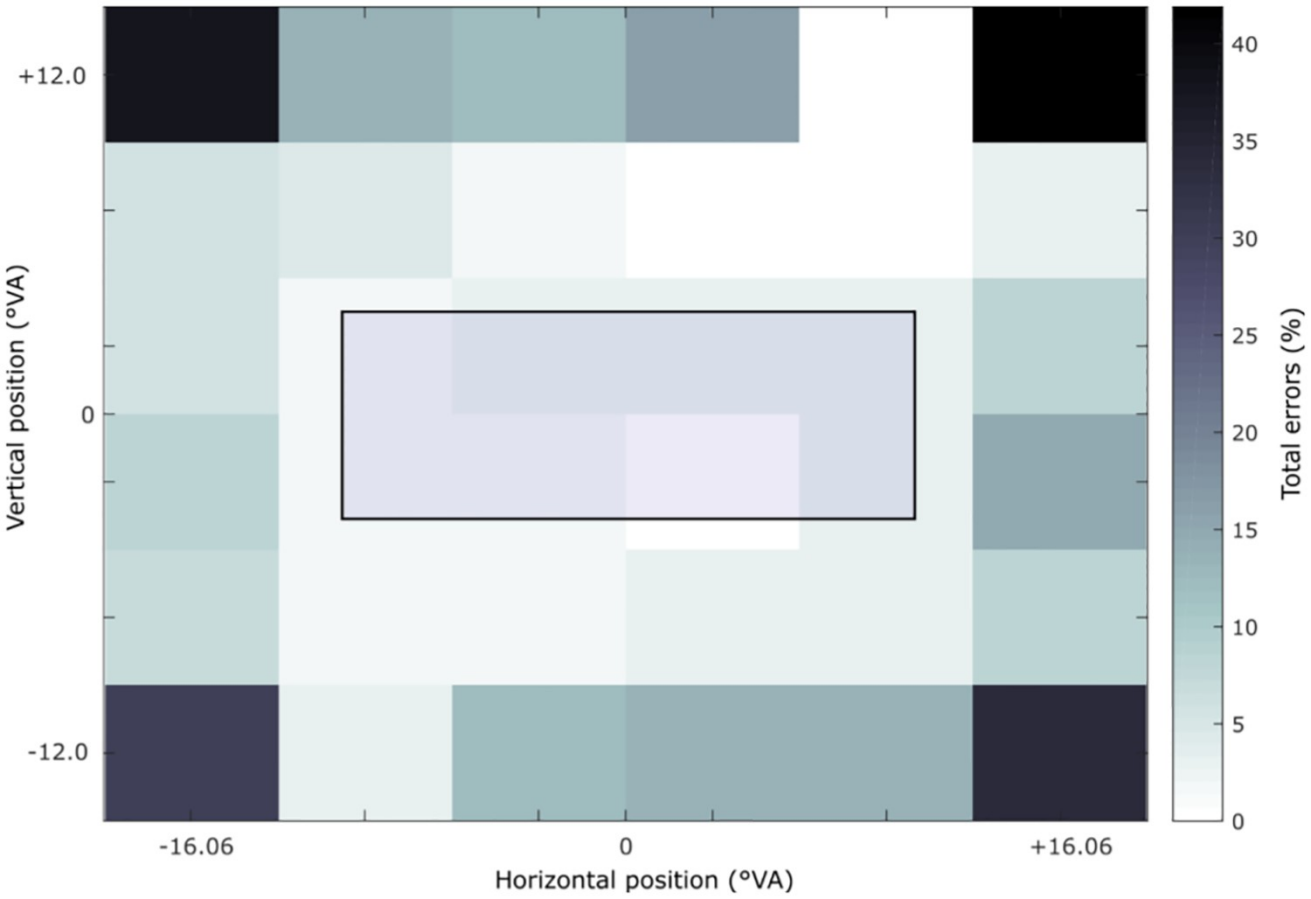
Overall percentage of detection errors for the visual acuity detection task. Each of the 6x6 squares displays the error rate across participants made at this position (reflecting the screen and visual field of the participant). The colour bar on the right side indicates the percentage of errors made, ranging from no errors to a 50% detection error rate. Most errors were situated in the periphery. The blue square in the centre indicates the region of interest, where the different spatial tasks were presented (LM, MLB, GRA, GREY, LVD). Error rates ranged from 0–5% on average in this area.

Results of the visual acuity screening [27] showed that participants made most detection errors in the periphery (see Fig 2), with most extreme values at the outer corners, ranging from 30%– 42% (M = 35.8%; S.D. = 5.2) both within and between participants. Most importantly, in the middle of the visual field that corresponded to the area where the experimental stimuli were presented, accuracy (detection rate) during visual acuity testing was M = 98.2%; S.D. 1.18 (across 16 positions, excluding the outer positions). Collapsed across all 36 positions, detection accuracy was at an average of M = 91.25%; S.D. = 10.91. No single participant fell below this.

### Spatial biases

#### Manual line bisection task (MLB)

A one-sample t-test over all positions and participants displayed a significant leftward bias (pseudoneglect), t(36) = -2.73, p = .01 (*M* = -.64, S.D. = 1.42).

Day 1: t(36) = -2.48, p = .02, M = -.65; S.D. = 1.59, Day 2: t(36) = -2.57, p = .02, M = -.63, S.D. = 1.48.

#### Landmark (LM), Greyscale (GREY) and Gratingscale (GRA) task

There was no bias identified for the LM task, on either testing day, or when collapsed together Day 1: t(36) = -.65, p = .52, M = -.08; S.D. = .78,

Day 2: t(36) = -.51, p = .61, M = -.05; S.D. = .64, Average of testing days: t(36) = -.66, p = .51, M = -.07, S.D. = .65. However, the GREY task showed a significant leftward bias (pseudoneglect) on each testing day and when averaged across testing days.

Day1: t(36) = -2.23, p = .03, M = -1.05; S.D. = 2.86, Day 2: t(36) = -2.13, p = .04, M = -.92; S.D. = 2.62, Average of testing days: t(36) = -2.65, p = .01, M = -.95, S.D. = 2.19.

The GRA task showed no significant bias to either side across testing days.

Day 1: t(36) = 1.30, p = .20, M = .37, S.D. = 1.73;

Day 2: t(36) = 1.07, p = .29, M = .31, S.D. = 1.75;

Average of testing days: t(36) = 1.27, p = .21., M = .31; S.D. = 1.49.

### Lateralised visual detection task (LVD)

#### D-Prime

Collapsed over both testing days, participants at a group-level correctly rejected 95% of catch trials and correctly identified an average of 40% of the presented targets in LVF and 37% in the RVF. The lateralisation index, which was collapsed over both testing days *d*′*RVF* − *d*′*LVF* = *d*′(*group bias*) was M = -.05, and did not support a lateralised bias in older adults when tested against zero [Day 1: t(35) = -1.04, p = .31, M = -.07; S.D. = .38, Day 2: t(35) = -.23, p = .82, M = -.02; S.D. = .50, Average of both days: t(35) = -.65, p = .52, M = -.05; S.D. = .42].

#### LVD (PF 50%)

A one-sample t-test against zero on the lateralisation index *RVF* − *LVF* = *PSE*(*group bias*), did not support a lateralised bias. Day 1: t(35) = 1.81, p = .08, M = .46; S.D. = 1.51, Day 2: t(35) = 1.69, p = .10, M = .45; S.D. = 1.58, Average of both testing days t(35) = 1.33, p = .19, M = .30; S.D. 1.36. Furthermore, a 2 (Side) x 2(Day) Repeated Measures ANOVA (PF 50%) revealed no differences across LVF vs. RVF positions and testing days. No main effect for day F(1, 35) = .18, p = .67 or side F(1, 35) = .97, p = .33, and no interaction F(1, 35) = .02, p = .90.

### Overall task bias summary

**Fig 3:**
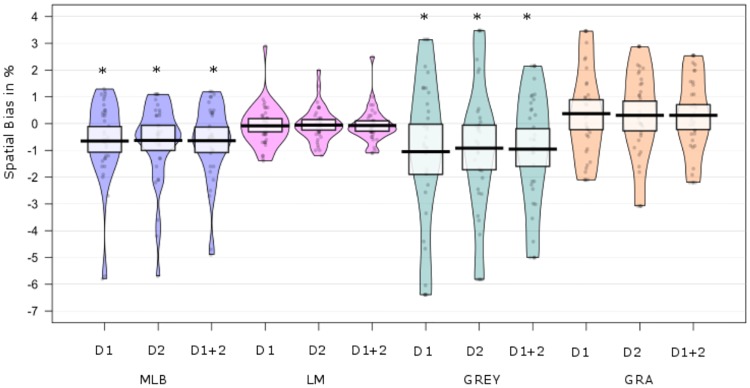
Overall spatial bias for the MLB, LM, GREY and GRA tasks. The spatial biases are shown for testing Day 1, Day 2 and collapsed over both testing days. Values below zero indicate leftward bias, while values above zero indicate a rightward bias. The violin plots are overlaid with the raw data of the spatial biases per individual to show the distribution of PSE in % of total line length. Boxplots display the group level mean and 95% HDI. Significant values compared to 0 are marked with an asterisk (*). Only the MLB and GREY tasks displayed a significant bias.

**Fig 4:**
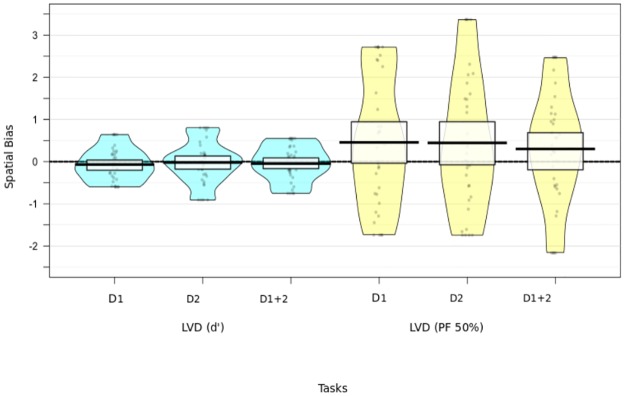
Overall spatial bias for LVD Task across testing days. The violin bars show the mean spatial bias, and the individual biases are overlaid for Day 1, Day 2 and the mean for both testing days across the LVD task and its two analyses (D’Prime and PF50%). Group level means are displayed as the black bar within the box displaying 95% HDI. None of the biases were significant.

In brief, across the five spatial measures, only the MLB and GREY task showed a significant leftward spatial bias (pseudoneglect) in older adults (see Fig 3). The GRA, LM, (see Fig 3) and LVD task (see Fig 4) did not show a lateralised bias on either testing day in older participants.

### Intra-task reliability

**Fig 5:**
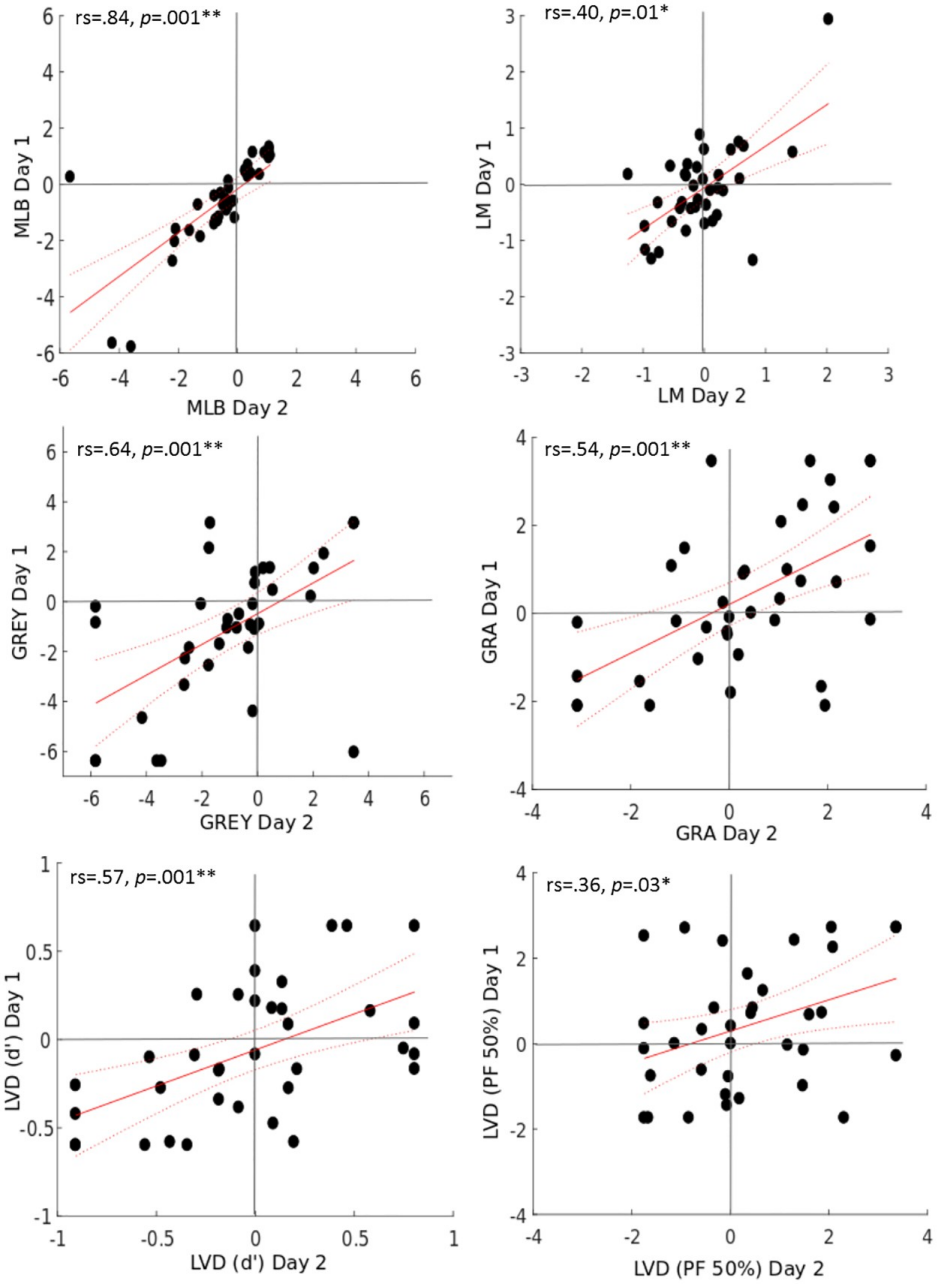
Intra-task correlation plots. Plots showing the MLB, LM, GREY, GRA and LVD tasks with D’Prime and PF50% analysis between Day 1 and Day 2. The red line displays the linear best fit and the dotted lines display the 95% confidence bounds.

In order to assess the test-retest reliability between both testing days, the five spatial tasks were analysed separately with a series of Spearman rho correlations on the biases obtained on Day 1 versus Day 2 (see Fig 5). Results showed significant correlations between both testing days on all tests (illustrated in Table 1).

**Table 1:** Intra-task correlations.

	*MLB*	*LM*	*GRA*	*GREY*	*LVD (d’)*	*LVD (PF 50%)*
*95% CI*	rs = .84	rs = .40	rs = .54	rs = .64	rs = .57	rs = .36
	p = .001**	p = .01*	p = .001**	p = .001**	p = .001**	p = .03*
Lower	.61	.06	.23	.32	.25	-.02
Upper	.96	.70	.78	.87	.80	.66

Results of the Spearman’s rho (rs) correlation and p- value for the 5 spatial tasks tested between two testing sessions.

Significant correlations at α = .01, corrected for multiple comparison are marked with double asterisk (**).

α = .05 are marked with a single asterisk (*).

### Inter- task reliability

Following on from the confirmed test-retest reliability of the tasks, Spearman’s rho correlations were used to investigate whether the magnitude of spatial bias in one task was correlated with the magnitude of spatial bias in the other 4 tasks. The results are illustrated in Table 2.

**Table 2:** Inter-task correlations.

	*LM*	*GRA*	*GREY*	*LVD (d’)*	*LVD (PF 50%)*
*N*	37	37	37	36	36
*MLB*	rs = .07	rs = -.06	rs = -.14	rs = .08	rs = -.001
	p = .70	p = .73	p = .42	p = .65	p = .98
95% CI Lower	= —.31	= -.44	= —.44	= -.23	= -.30
95% CI Upper	= .42	= .31	= .26	= .40	= .32
*LM*		rs = .27	rs = .36	rs = -.26	rs = .19
		p = .11	p = .03	p = .13	p = .27
95% CI Lower		= -.12	= -.54	= -.54	= -.21
95% CI Upper		= .60	= .08	= .08	= .522
*GRA*			rs = .36	rs = .01	rs = .03
			p = .03	p = .95	p = .85
95% CI Lower			= .02	= -.35	= -.35
95% CI Upper			= .65	= .35	= .45
*GREY*				rs = .14	rs = -.004
				p = .42	p = .98
95% CI Lower				= -.16	= -.37
95% CI Upper				= .43	= .34
*LVD (d’)*					rs = -.71**
					**p = .001**
95% CI Lower					= -.92
95% CI Upper					= -.45

This table shows the results of the Spearman’s rho (rs) correlation and p- value for the 5 spatial tasks tested.

Significant correlations at α = .003, corrected for multiple comparison are marked with an asterisk (**)

**Fig 6:**
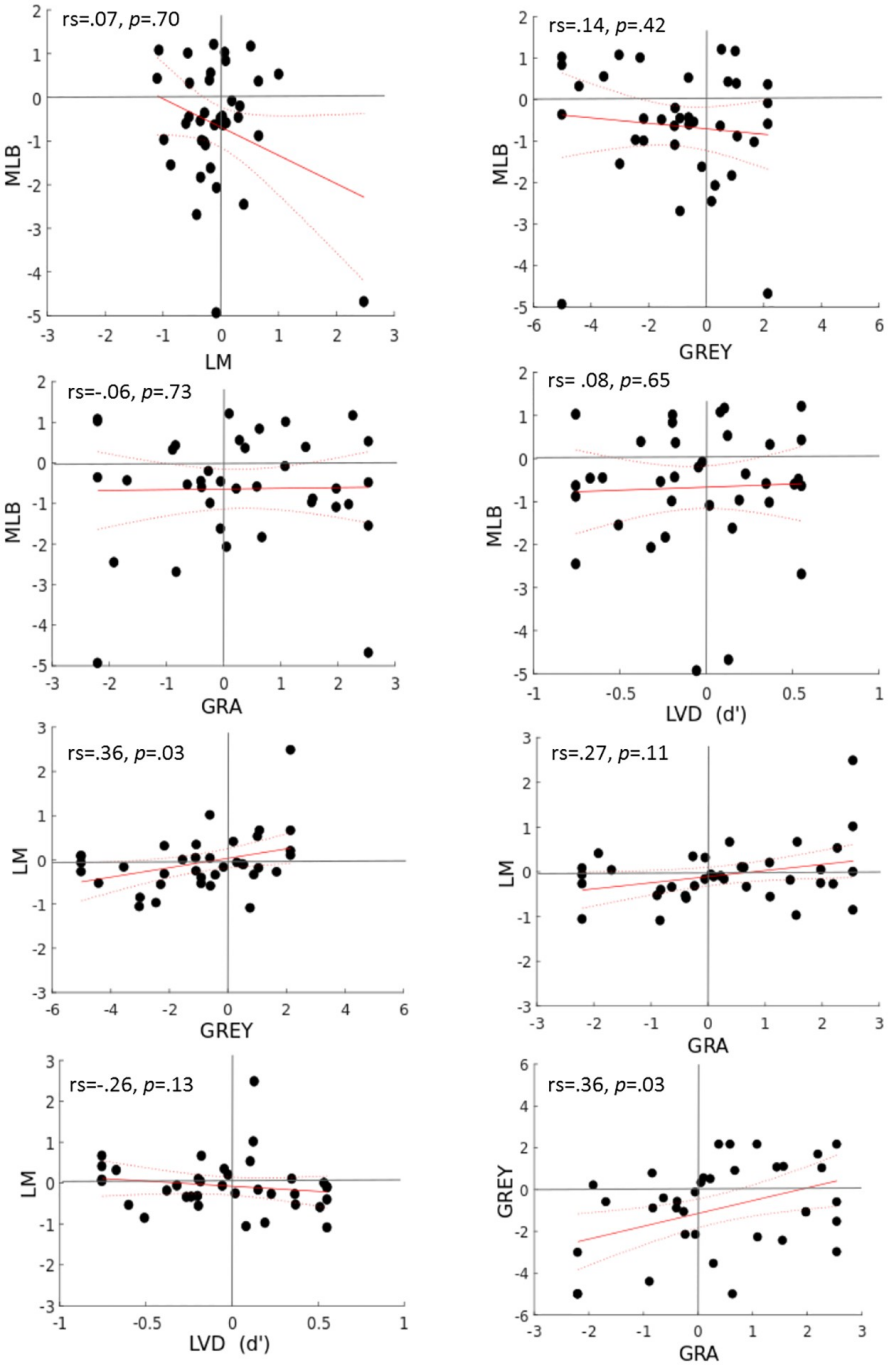
Inter- task correlation plots for MLB, LM, GREY and GRA task.

**Fig 7:**
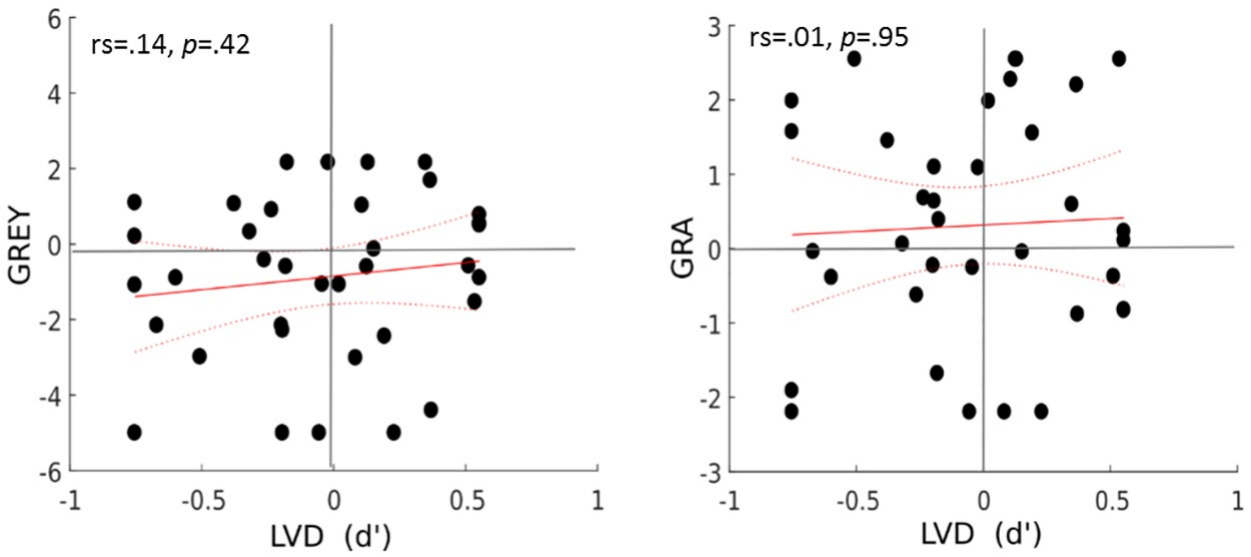
Inter- task correlation plots for LVD task. Plots showing the correlation of the spatial tasks with each other MLB, LM, GREY, GRA and LVD task with D’Prime across the mean of both testing days. The red line displays the linear best fit and the dotted lines display the 95% confidence bounds.

The results showed that collapsed over both testing days, only the LVD task measures of D’Prime and PF 50% were strongly correlated (rs = .71, p = .001), confirming the reliability of the analysis methods. The LM and GREY as well as the GRA and GREY task showed a weak correlation, which did not survive Bonferroni correction. No other tests were correlated with each other (see Figs 6 and 7).

### Task precision (curve width)

Curve width was analysed for the tasks LM, Grating-, Greyscales and LVD tasks as a measure of precision of task engagement and sustained attention over both testing days. High task precision is reflected in a steep curve.

Correlating the intra-task curve widths (spearman’s rho) over both testing days confirmed a consistency of precision between the testing sessions in older adults (LM rs = .62, p = .001, GRA rs = .63, p = .001, GREY rs = .67, p = .001, LVD Left visual field rs = .55, p = .001, Right visual field rs = .65, p = .001). Paired samples t-tests for Day 1 vs Day 2 showed that precision improved on the second day for the LM t(36) = -2.85, p = .007 (Day 1 M = -.89 Day, 2 M = -.709) and the GRA task t(36) = -2.04, p = .05 (Day 1 M = -6.36, Day 2 M = -5.41), as reflected in a narrower curve on the second day.

### Comparison between young and older adults

**Fig 8:**
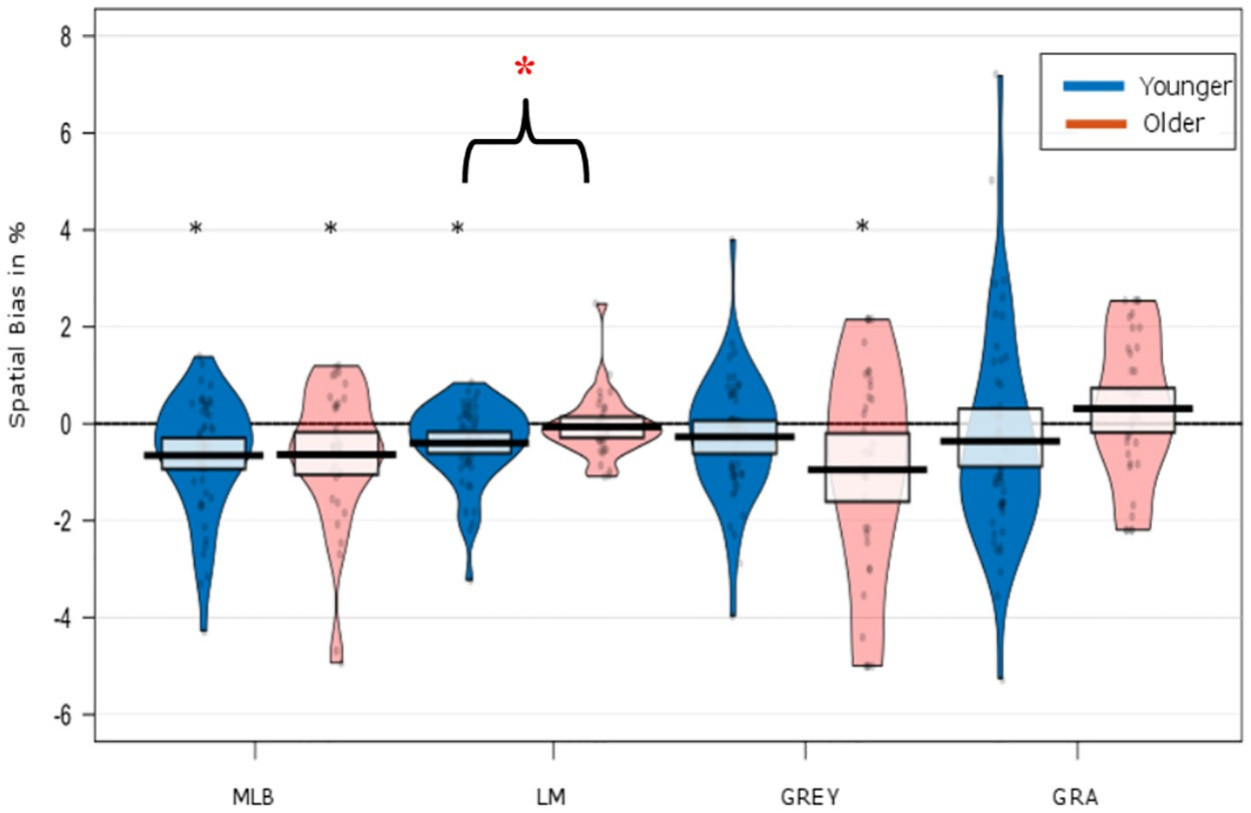
Spatial biases of the younger and older adults. The violin plots display the group mean spatial biases collapsed over both testing days for the four spatial tasks. The individual spatial biases are overlaid. The boxplots display the mean per task with 95% HDI. A significant spatial bias is depicted with a black asterisk (*). A significant difference between the age groups within a task is depicted with a bracket and a red asterisk (*).

Despite the slight difference in viewing distance between the two age group samples (present study vs. Learmonth, Gallagher *et al*.,[9,10]), four of the five spatial tasks were procedurally identical in the older group (N = 37) reported here, relative to the young adult sample investigated by Learmonth et al. [9,10](N = 50). We therefore performed a direct, between-group comparison between the young and older adults to assess age-related differences in spatial biases across the 4 tasks. A one-way MANOVA on the mean data obtained on Days 1&2 revealed a significant effect of age on spatial biases. F(4, 82) = 3.58, p = .01; *ηp*^2^ = .15, Wilks’ Δ = .85. The ANOVA revealed an interaction between LM x Age = F(1, 85) = 4.26, p = .04,; *ηp*^2^ = .05 but no other measures yielded a significant interaction. *Welch’s t-tests* revealed that the Landmark task elicited significantly different biases in young and older adults, t(84,6) = -2.14, p = .04). While young adults showed a leftward bias (M = -.39) (pseudoneglect), older adults had no mean spatial bias for this task (M = -.08), thus not showing pseudoneglect. All other measures did not differ significantly between age groups (see Fig 8).

#### Additional gender analyses for the MLB, LM, GREY and GRA tasks

We were encouraged to perform additional gender analyses as some previous research suggests stronger bias effects in males [16,17,36]. However, deviating findings have since been reported (see [37]) and Benwell et al. [24] reported no gender specific effects in the landmark task although age effects were present (see [24]).

Results of a series of 2(Age) x 2(Gender) ANOVAs run for each of the four spatial tasks (that we compared across age groups (MLB, LM, GREY, GRA, see Fig 8)) and tested on the mean spatial biases across both days, revealed no gender specific main effects nor interactions, suggesting that gender did not influence the magnitude of spatial bias within or between the age groups. (MLB: Age F(1,14) = 1.11, p = .31 Gender F(1,14) = .24, p = .63 Age x Gender F(1,14) = .003, p = .96. LM: Age F(1,14) = 1.24, p = .28 Gender F(1,14) = 1.25, p = .28 Age x Gender F(1,14) = .13, p = .72. GREY: Age F(1,14) = 2.82, p = .11 Gender F(1,14) = .77, p = .40 Age x Gender F(1,14) = .02, p = .90 GRA: Age F(1,14) = 2.73, p = .12 Gender F(1,14) = 2.23, p = .16 Age x Gender F(1,14) = .32, p = .58.

Nonetheless since our original design was not set up to investigate gender and/or age-related gender differences, these results should be treated with caution, especially since, although the older adult group was relatively gender balanced (18 male and 19 female participants), the young adult sample had a distribution of 35 females to 15 males only.

## Discussion

The present study investigated possible age-related changes in spatial asymmetry and its impact on the intra- and inter task reliability of five commonly used spatial tasks. Summing up the results, the 5 tasks presented here elicited consistent spatial biases in older adults when tested on different testing days, which contrasts with reports of higher variability in test-retest reliability in older participants [38]. Overall, the manual line bisection and greyscales tasks elicited significant, stable leftward biases (pseudoneglect), whereas the remaining tasks did not reveal a significant spatial asymmetry (LM, GREY, GRA). Secondly, no relationship was found between any of the tasks, when investigating the inter-task correlations, replicating the results reported for young adults by Learmonth et al. [9,10]. Finally, a direct comparison between the young and older adult datasets indicated that the landmark task separated the groups in terms of spatial bias, with young adults demonstrating pseudoneglect that was not present in the older group.

### Intra-task correlations

Encouragingly, the stable test-retest reliability for each of the five measures of spatial attention that we previously reported for young adults [9,10] were also found in the present, older age group. This seems to support the conclusion by Learmonth et al. [9,10] that each of the five tasks tested here activate a consistent property of the attention network, each dependent on the respective task-demands at hand.

### Leftward biases in older adults

Interestingly, none of the tasks tested here displayed a critical rightward bias in older adults. On the contrary, similarly to Brooks et al.[8], we demonstrated that a leftward bias was present in two tasks: we replicated the previously reported leftward bias for line bisection [8,36,37], as well as for the greyscales task, which had been found to elicit strong leftward biases in very elderly adults aged 80–89, as well as young adults from 18–29 years [7]. These findings seem to support prior claims that right hemispheric specialization for attentional orienting can be retained into old age [8]. However, here we add to this literature by identifying that this may only hold true for certain visuospatial tasks. The current results are in line with our previous work, where we have argued that different spatial attention tasks place a unique set of cognitive and motor demands on the spatial attention networks [9,10,39]. Here, we have replicated this finding within a healthy older sample and tentatively suggest that the neural resources that are involved in the manual line bisection and greyscales tasks may be less susceptible to age-related neural changes.

### Presence of ageing effects

In a direct comparison with the young adult sample of Learmonth et al. [9,10], we found that only the landmark task was modulated by ageing (i.e. a leftward bias in young adults and no significant bias in the older group). The attenuated spatial bias in the older adults here could potentially reflect a selective age-related decline of right-hemispheric processes that are involved in undertaking this task (e.g. as described by the Hemispheric Asymmetry Reduction in Older Adults (HAROLD) model of cognitive aging [40]). In fact, our group recently reported evidence for decreased right hemisphere activity in older adults when performing the landmark task in EEG recording [27]. Similar to the present results, that sample of older adults also showed an absence of leftward bias in comparison to young adults. In addition, there was a time-window of right-sided lateralisation of neural activity in young adults, which was absent in the older group. It may be that, in line with the HAROLD model, the older participants recruited supplementary contralateral brain areas when dealing with this task to maintain their performance (for a discussion see [27,40,41]. In fact, all of the current participants were within normal range on the MOCA test, which is further evidence that this observed shift is likely to be attributed to healthy neuronal mechanisms of aging, rather than cognitive decline. However, the difference in behavioural performance between the groups was subtle, with a substantial overlap in the range of biases in both young and older adults (see Fig 8). The specific mechanism, and indeed the relevance of this age-related shift in the landmark task must now be clarified.

### Inter-task correlations

The spatial tasks showed no significant correlation between each other, even though they were procedurally similar. Although the GREY and GRA tasks and the LM and GREY tasks showed small between task correlations, these did not survive Bonferroni correction. Most relevant to this, a recent study by Agnew, Phillips and Pilz [42] also reported an absence of correlation between attention tasks and biological motion processing both in young and older adults (while observing age related slower responses in older adults overall). Furthermore, a different study [43] again reported only weak correlation between a battery of visual tasks for older and young adults, and the authors further emphasize an absence of a common underlying factor that would explain the visual abilities from age effects [43]. So on the basis of our earlier results in young adults [9,10], and findings in the past and very recent literature [11–13,42,43], this lack of between task correlations appears to be independent of age. This poses a substantial problem for the assessment of spatial attention, as any attempt to generalise spatial bias effects across tasks is not merited and should be treated with caution.

### Specific task effects

Despite their procedural similarity, the landmark (LM) and manual line bisection (MLB) task yielded different spatial biases, with leftward biases for the MLB and an absence of spatial bias for the LM task. Both measurements have been found previously to engage right lateralized brain activity and involve the right intra-parietal sulcus (IPS) and right lateral peristriate cortex [44], and in our younger sample we found directional effects to be the same [9,10]. Nonetheless two studies have now reported a lack of correlation between these two tasks in young adults [9,10,44] and together with our reported findings in this older sample, equivalence between these tasks can no longer assumed to be a given.

For the first time, we tested older adults on the Gratingscale (GRA) task, which is procedurally similar to the Greyscale (GREY) task. Again, as for LM vs MLB, we report divergent spatial biases with significant pseudoneglect for the GREY task but no significant spatial asymmetry in the GRA task. Both results resemble the spatial bias observed in young adults in Learmonth et al.[9,10], who reported no bias for the GRA task and also a significant leftward bias on day one for the GREY task, which then attenuated at re-test. These findings were somewhat contrary to findings of a robust preference in young adults for the left visual field in both tasks (GRA, GREY), as well as an intra-task correlation between them [28]. It is worth noting that in the GRA task here, participants assessed the number of stripes, directing their attention to the largest amount of “thin stripes”. Low spatial frequencies (SFs) are processed in the left hemisphere, while high SFs are more likely processed in the right hemisphere [28,45]. With the focus on the high SF (more “thin stripes”), the right hemisphere should be more involved in spatial judgements, leading to the observed leftward bias and confirming the claims made by Niemeier et al.[28] for a robust measure of spatial bias. Our finding of an absence of a leftward bias for older adults, could reflect the finding that older adults become less sensitive to spatial frequency processing [46–49], so while the task may not have been sensitive enough for young adults, it might have been sufficed to elicit a spatial shift in the older sample.

Finally, the results of the LVD task did not elicit a significant bias in older adults, confirming earlier findings of an absent bias [10,27]. Yet we have to concede that despite adjustments in the design to ensure an adequate stimuli range for older participants, which included 10 different stimulus sizes, the task difficulty varied vastly among these older adults, with some perceiving the task as very difficult (or others not challenging enough).

### Is pseudoneglect purely a measure of spatial attention? Limitations

In light of the directional variability of the spatial biases reported here and in recent studies (which also seem largely independent of age), it is probable that both the direction and magnitude of pseudoneglect are dynamically modulated by a range of task- and participant-dependent variables, rather than reflecting a fixed measure of laterally biased spatial attention *per se*. Differences in task difficulty across the spatial tasks might drive the observed presence and absence of leftward bias. The same could apply for differences in sustained attention [50] both within and across tasks. An increase in task difficulty for example could result in an attenuation of a leftward bias but this might be driven by individual rather than age-related differences. As a consequence, a separation into age groups and a discussion of age related changes in pseudoneglect may not be meaningful without first quantifying these additional factors to understand how they might interact. At present we do not know, so it seems paramount now to understand each spatial task we currently use, with respect to possible other modulating influences. For example, we are currently investigating whether an increase in attentional load affects lateralised visual detection. We expect faster left dot detection to be attenuated under high attentional load but this predicted attenuation may (or may not) be modulated further by age. A similar attempt in this direction has been made in a very recent study by Chen et al. [51] who reported that adding visual noise to landmark, greyscale and gratingscales tasks increased left spatial bias in young adults. Further neuroimaging studies should also be undertaken to characterize such possible modulators of pseudoneglect.

As a final, if unrelated, caveat (and not the main focus of our study), although we took great care in ensuring that the older adults perceived the stimuli accurately a) by testing acuity with the Snellen chart (and all participants were perfect) and b) by carrying out further visual field visual acuity screening, with older adults performing with above 98% accuracy for the visual area all stimuli were presented in, it is still possible that the acuity of the younger adults was better and that that may account for some age-related differences (although we deem this unlikely).

## Conclusion

Similar to our earlier findings in young adults, this study reports good test- retest reliability of five spatial measures taken over different days, in a population of cognitive healthy older adults. We confirm that the direction of the spatial bias is task sensitive rather than task general. The MLB and GREY tasks showed a spatial bias to the left side of space, with older adults demonstrating pseudoneglect for these tasks. In a direct comparison to young adults, only the LM task showed an attenuated spatial bias in older compared to younger adults, possibly consistent with neuronal findings of an age-related reduction of the right hemisphere [27]. As our participants did not score clinically on measures of early neurological decline, this is likely a result of healthy aging. Yet, the inconsistent bias results found here and in the more recent literature seem to point to other factors possibly driving these effects. An increase in task difficulty or sustained attention for example, could account for the absence of leftward biases described here (and these modulators could be age dependent or not). Fitting with this assumption are the (now repeatedly observed) lack of inter-task correlations. This complicates generalisation and comparability of pseudoneglect effects across different tasks, age-groups and hence studies.

## Supporting information

S1 Table The table details the individual spatial biases per spatial task on testing days 1 and 2, as well as averaged across both testing days. (XLSX)
